# The Cost of Trained Nurses

**Published:** 1892-10-08

**Authors:** 


### Passing Topics.

### THE COST OF TRAINED NURSES.

There is an amazing amount of reason in most arguments when they harmonise with our feelings, or when we examine them too exclusively from one point of view. The correspondents who are complaining about the cost of employing a trained nurse have a great deal of right upon their side, because we are being continually told, and told truly, that good nursing is of the first importance in serious illness. Some people go so far as to hint—though here the truth is not quite so patent—that the nurse is of more importance than the doctor; but however that may be, we are led to the conclusion that the sufferer who has unskilled nursing commands a very much diminished chance of recovery. In this way, good nursing is most effectually proved to be a necessity of suffering humanity. But if a necessity, it ought to be brought within the reach of all. It is useless to prove to us that we ought to possess something, and must possess it, and then, after convincing us we cannot do without that something, to set a prohibitive price upon it, as if it were merely a luxury.

That the cost of a trained nurse is prohibitive to the large majority of householders is amply demonstrated by the figures given. We say *a* trained nurse, but the nurses sent out by institutions very often go in couples, and a nurse for the day and another for the night constitute a common provision for one patient. The actual outlay is set out by a correspondent thus: Two guineas a week for each nurse; half-a-crown each for beer-money—surely rather a humiliating entry; and another half-crown each for laundry; making £4 14s. per week in wages, "besides the good living," and as we suppose, the lodging.

On the nurse's side we have to remember that a considerable period has been spent by her, and much trying work has been gone through, in acquiring a full and sufficient knowledge of the art she practises, that the public now look for a certain amount of education and refinement in a nurse, and that the very fact of the quality of our nurses is improved, renders it only right an increased value should be set upon their services.

Undoubtedly it is difficult to reconcile the two requirements—efficiency and cheapness. Practically the difficulty is insurmountable. Even if a much larger number of women than is likely, could be attracted into the nursing ranks, and if steps could be taken to stay the heavy depletion which arises from a variety of causes, the ever-increasing demand for trained nurses would ensure the maintenance of a fair standard of remuneration. We see no way of regulating the cost of nurses by any other than the ordinary rules of supply and demand, and as the supply will in all probability never prove superabundant, the people of slender means will have to be content in respect of nursing as of many other good things of this life, to leave it to be enjoyed by those whom fortune has more amply favoured.

Only one suggestion seems open to us, and that is that no purely artificial and needless addition should be made to the burden of sickness. The desire at headquarters to make a profit is now a little too obvious. Cases have occurred where the employment of two nurses has been insisted upon unnecessarily. Doctors never, and other workers in the world's hive not always can ensure undisturbed periods of daily rest and recreation. The avocation of a sick nurse is one which particularly calls for an ability and willingness to adopt oneself to circumstances, and while we all know there are thoughtless, exacting, and unscrupulous employers to be guarded against, we do not think that the rules enforced here and there ostensibly in the interest of the nurses are calculated either to add to the dignity of the calling or to enlarge its usefulness. Agricola.

